# A summary of the ComParE COVID-19 challenges

**DOI:** 10.3389/fdgth.2023.1058163

**Published:** 2023-03-08

**Authors:** Harry Coppock, Alican Akman, Christian Bergler, Maurice Gerczuk, Chloë Brown, Jagmohan Chauhan, Andreas Grammenos, Apinan Hasthanasombat, Dimitris Spathis, Tong Xia, Pietro Cicuta, Jing Han, Shahin Amiriparian, Alice Baird, Lukas Stappen, Sandra Ottl, Panagiotis Tzirakis, Anton Batliner, Cecilia Mascolo, Björn W. Schuller

**Affiliations:** ^1^ Department of Computing, Imperial College London, London, United Kingdom; ^2^ Department of Computing, FAU Erlangen-Nürnberg, Erlangen-Nürnberg, Germany; ^3^ Institute of Computer Science, Universität Augsburg, Augsburg, Germany; ^4^ Department of Computer Science and Technology, University of Cambridge, Cambridge, United Kingdom; ^5^ Department of Computing, University of Southampton, Southampton, United Kingdom

**Keywords:** COVID-19, machine learning, Digital Health, computer audition, deep learning

## Abstract

The COVID-19 pandemic has caused massive humanitarian and economic damage. Teams of scientists from a broad range of disciplines have searched for methods to help governments and communities combat the disease. One avenue from the machine learning field which has been explored is the prospect of a digital mass test which can detect COVID-19 from infected individuals’ respiratory sounds. We present a summary of the results from the INTERSPEECH 2021 Computational Paralinguistics Challenges: COVID-19 Cough, (CCS) and COVID-19 Speech, (CSS).

## Introduction

Significant work has been conducted exploring the possibility that COVID-19 yields unique audio biomarkers in infected individuals’ respiratory signals (1–14). This has shown promising results although many still remain sceptical, suggesting that models could simply be relying on spurious bias signals in the datasets (15, 12). These worries have been supported by findings that when sources of bias are controlled, the performance of the classifiers decreases (16, 17). Along with this, cross dataset experiments have reported a marked drop in performance when models trained on one dataset are then evaluated on another dataset, suggesting dataset specific bias (18).

Last summer, the machine learning community were called upon to address some of these challenges, and help answer the question whether a digital mass test was possible, through the creation of two COVID-19 challenges within the Interspeech Computational Paralinguistics challengE (ComParE) series: COVID-19 Cough, (CCS) and COVID-19 Speech, (CSS) (19). Contestants were tasked to create the best performing COVID-19 classifier from user cough and speech recordings. We note that another COVID-19 detection from audio challenge was run at a similar time to ComParE, named DiCOVA (20), and point the inquisitive reader to their blog post^1^ which details a summary of the results.

## Challenge methodology

Both COVID-19 cough and speech challenges were binary classification tasks. Given an audio signal of a user coughing or speaking, challenge participants were tasked with predicting whether the respiratory signal came from a COVID-19 positive or negative user. After signing up to the challenge, teams were sent the audio files along with the corresponding labels for both the training and development set. Teams were also sent the audio files from the test set without the corresponding labels. Teams were allowed to submit five predictions for the test set from which the best score was taken. The number of submissions was limited to avoid overfitting to the test set.

**Figure 1:**
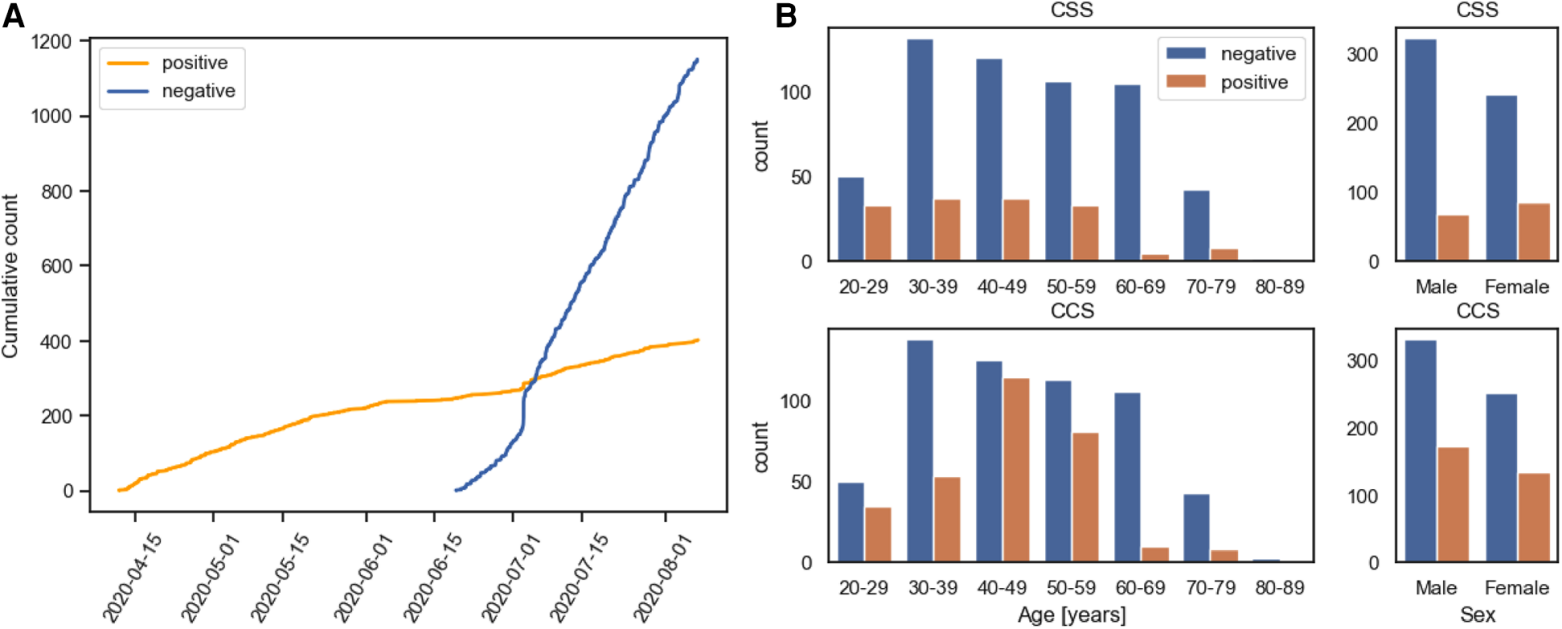
(**A**) Is a cumulative plot detailing when COVID-19 positive and negative submission to both the CCS and CSS were made. (**B**) Details the age and sex distribution of COVID-19 positive and negative participants for the CCS and CSS Sub-Challenges.

The datasets used in these challenges are two curated subsets of the crowd sourced Cambridge COVID-19 Sounds database (1, 21). COVID-19 status was self-reported and determined through either a PCR or rapid antigen test, the exact proportions of which are unknown. The number of samples of both positive and negative cases for these selected subsets are detailed in Table 1. The submission date for both COVID-19 positive and negative case recordings are detailed in Figure 1A. Figure 1B shows the age distribution for both CSS and CCS challenges.

**Table 1:** ComParE COVID-19 sub-challenges dataset splits. Values specify the number of audio recordings. We note that disjoint participant train, development, and test splits were ensured.

	CCS${}^{a}$			CSS${}^{b}$		
	Train	Dev	Test	Train	Dev	Test
COVID-19-positive	71	48	39	72	142	94
COVID-19-negative	215	183	169	243	153	189
Total	286	231	208	315	295	283

${}^{a}$CCS – COVID-19 Cough Sub-Challenge.

${}^{b}$CSS – COVID-19 Speech Sub-Challenge.

## Overview of methodologies used in accepted papers at interspeech 2021

**Figure 2:**
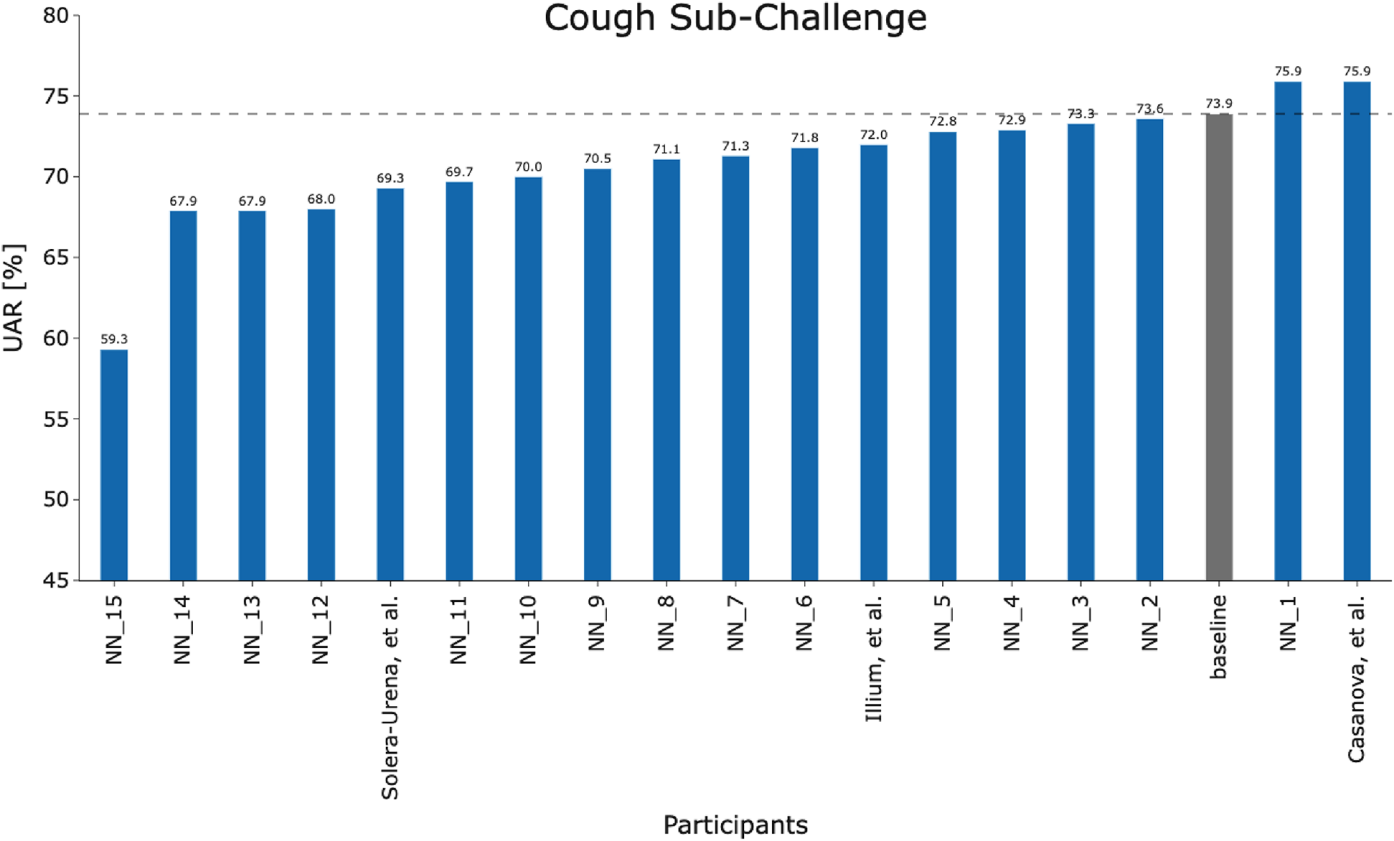
Team performance on the held out test set for the COVID-19 Cough Sub-Challenge.

Last year, 44 teams registered in both the ComParE COVID-19 Cough Sub-Challenge (CCS) and the COVID-19 Speech Sub-Challenge (CSS) of which 19 submitted test set predictions. Five of the 19 teams submitted papers to INTERSPEECH which were then accepted. Results for both CCS and CSS were reported in two of these papers, while two papers reported results exclusively for CCS and one paper exclusively for CSS. In this section, we provide a brief overview of methodologies used in these accepted works which included data augmentation techniques, feature types, classifier types, and ensemble model strategies. Teams that did not have their work accepted at INTERSPEECH 2021 will be named NN_X to preserve anonymity. NN refers to *nomen nescio* and X is the order in which they appear in Figure 2. The performance measured in Unweighted Average Recall (UAR) achieved by these methodologies is summarised in Table 2; UAR has been used as a standard measure in the Computational Paralinguistics Challenges at Interspeech since 2009 (26). It is the mean of the diagonal of the confusion matrices in percent and by that, fair towards sparse classes. Note that UAR is sometimes called “macro-average,’ see (27).

**Table 2:** Summary of methodologies used in accepted papers at Interspeech 2021 along with their classification performance. Unweighted Average Recall (UAR) and Unweighted Average F1 (UF1) metrics are provided [%].

Team name	Data Aug.	Feature type	Classifiers	Ensemble	Cough		Speech	
					UAR	UF1	UAR	UF1
Solera-Urena et al. (22)	✗	TDNN-F, VGGish, PASE+	SVM	✓	69.3	65.2	–	–
Casanova et al. (23)	✓	MFCC, mel-spectrogram	SpiraNet, CNN14, ResNet-38, MobileNet	✓	**75.9**	69.6	70.3	71.0
Klumpp et al. (24)	✓	mel-spectrogram	CNN, LSTM, SVM, LR	✗	–	–	64.2	64.3
Illium et al. (25)	✓	mel-spectrogram	Vision transformer	✗	72.0	71.1	–	–
Baseline (19)	✗	openSMILE, openXBOX, DiFE, DeepSpectrum, auDeep	SVM, End2You	✓	73.9	–	**72.1**	–

### Data augmentation

To combat the limited size and imbalance of the Cambridge COVID-19 Sounds database, the majority of the teams used data augmentation techniques in their implementation. Team Casanova et al. exploited a noise addition method and SpecAugment to augment the challenge dataset (23). Team Illium et al. targeted spectrogram-level augmentations with temporal shifting, noise addition, SpecAugment and loudness adjustment (25). Instead of using a data augmentation method to manipulate the challenge dataset, team Klumpp et al. used three auxiliary datasets in different languages aiming their deep acoustic model to better learn the properties of healthy speech (24).

### Feature type

The teams chiefly used spectrogram-level features including mel-frequency cepstral coefficients (MFCC) and mel-spectrograms. For higher-level features, the teams used the common feature extraction toolkits openSMILE (28), openXBOX (29), DeepSpectrum (30), and auDeep (31), where a simple support vector machine (SVM) model was built on top of these features. Team Solera-Urena et al. exploited transfer learning to extract feature embeddings by using pre-trained TDNN-F (32), VGGish (33), and PASE+ (34) models with appropriate fine-tuning on the challenge dataset. Team Klumpp et al. targeted to extract their own phonetic features by using an acoustic model consisting of convolutional neural network (CNN) and long short-term memory (LSTM) parts.

### Classifier type

Team Solera-Urena et al. (22) and the challenge baseline (19) fitted a SVM model to high level audio embeddings extracted using TDNN-F (32), VGGish (33), and PASE+ (34) models, and the openSMILE framework (28), respectively. While the challenge baseline (19) searched for the complexity parameter of the SVM ranging from $10^{-5}$ to 1, team Solera-Urena et al. (22) explored different kernels (linear, RBF), data normalisations (zero mean and unit variance, [0,1] range) and class balancing methods (majority class downsampling, class weighting). In addition to the SVM model, the baseline explored using the multimodel profiling toolkit End2You (35) to train a recurrent neural network using Gated Recurrent Units (GRUs) with hidden units of 64. Team Casanova et al. (23) utilised the deep models: SpiraNet (36), CNN14 (37), ResNet-38 (37), and MobileNetv1 (37) where they explored kernel size, convolutional dilatation, dropout, number of fully connected layer neurons, learning rate, weight decay and optimizer. Team Klumpp et al. (24) trained SVM and logistic regression (LR) models to perform COVID-19 classification on top of phonetic features extracted by their deep acoustic model. They explored the complexity parameter of the SVM ranging from $10^{-4}$ to 1. Team Illium et al. (25) adapted a vision transformer (38) for mel-spectrogram representations of audio signals. Tree-structured Parzen Estimator-algorithm (TPE) (39) was exploited in (25) for hyperparameter search mainly exploring embedding size, learning rate, batch size, dropout, number of heads and head dimension. The teams Solera-Urena et al., Casanova et al., and the baseline also reported classification results by using the fusion of their best features and classifiers. To conclude, Casanova et al. performed best among the accepted papers with a consistent performance over both CCS and CSS. This showed the importance of using proper data augmentation techniques and exhaustive exploration of deep models and hyperparameters for a transfer learning approach.

### Assessment of performance measures

Figure 4 visualises a two-sided significance test (based on a Z-test concerning two proportions, (40), section 5B) employing the CCS and CSS test sets and the corresponding baseline systems (19). Various levels of significance (α-values) were used for calculating an absolute deviation with respect to the test set, being considered as significantly better or worse than the baseline systems. Due to the fact that a two-sided test is employed, the α-values must be halved to derive the respective Z-score used to calculate the p-value of a model fulfilling statistical significance for both sides (40). Consequently, significantly outperforming the best CCS baseline system (73.9% and 208 test set samples) at a significance level of α=0.01 requires at least an absolute improvement of 6.7%; for CSS (best baseline system with 72.1% and 283 test set samples), the improvement required is 6.0%. Note that Null-Hypothesis-Testing with p-values as criterion has been criticised from its beginning; see the statement of the American Statistical Association in Wasserstein and Lazar (41) and Batliner et al. (42). Therefore, we provide this plot with p-values as a service for readers interested in this approach, not as a guideline for deciding between approaches.

Another way of assessing performance measures as for their “uncertainty” is computing confidence intervals (CIs). Schuller et al. (19) employed two different CIs: first, 1000× bootstrapping for test (random selection with replacement) and UARs based on the same model that was trained with Train and Dev; in the following, the CIs for these UARs are given first. Then, 100× bootstrapping for the corresponding combination of Train and Dev; the different models obtained from these combinations were employed to get UARs for test and subsequently, CIs; these results are given in second place. Note that for this type of CI, the test results are often above the CI, sometimes within and in a few cases below, as can be seen in (19); obviously, reducing the variability of the samples in the training phase with bootstrapping results on average in somehow lower performance. For CCS with a UAR of 73.9%, the first CI was 66.0%–82.6%; the second one could not be computed because this UAR is based on a fusion of different classifiers. For CSS with a UAR of 72.1%, the CIs were 66.0%–77.8% and 70.2%–71.1%, respectively. Both Figure 4 and the spread of the CIs reported demonstrate the uncertainty of the results, caused by the relatively low number of data points in the test set.

## Results and discussion

**Figure 3:**
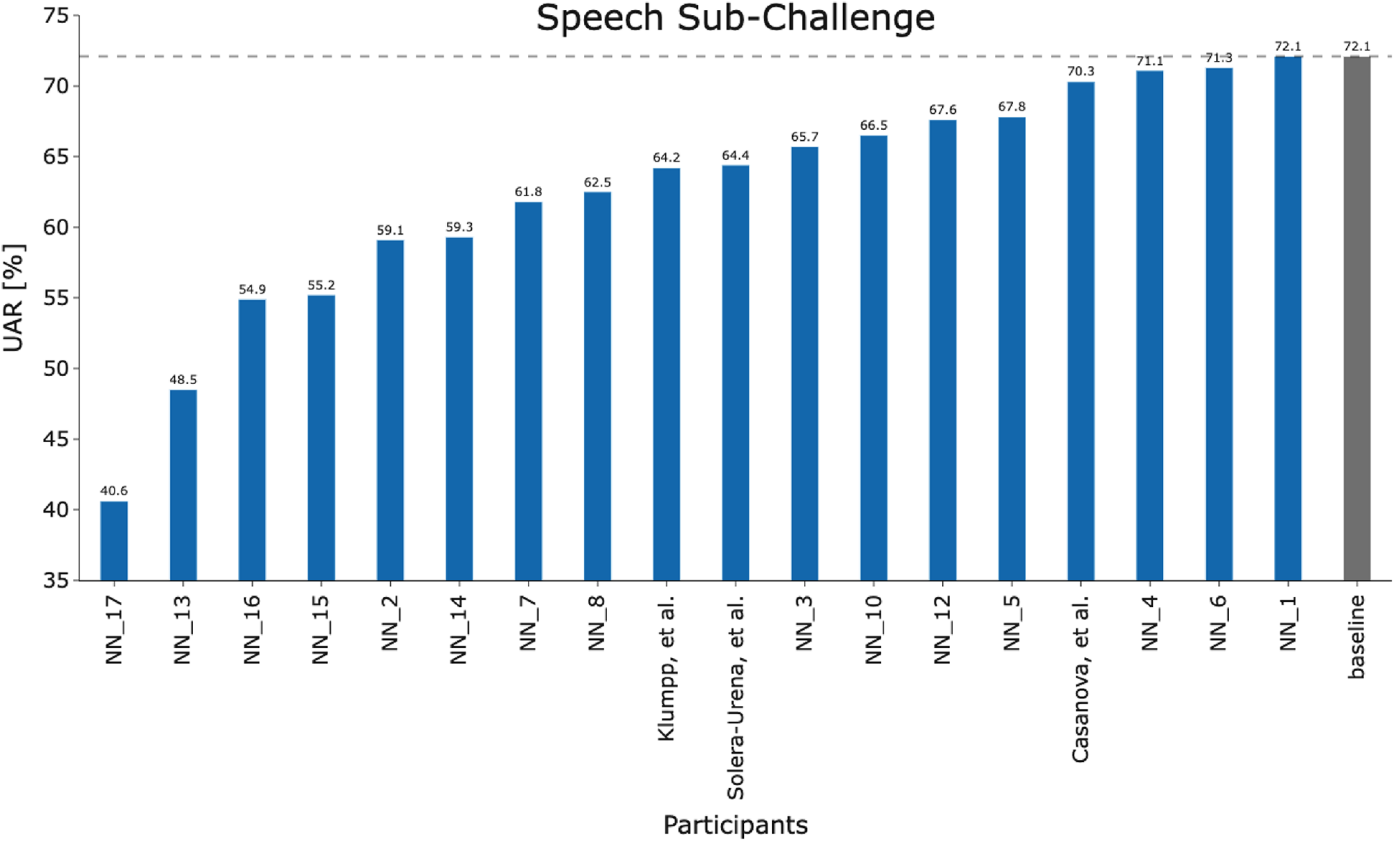
Team performance on the held out test set for the COVID-19 Speech Sub-Challenge.

**Figure 4:**
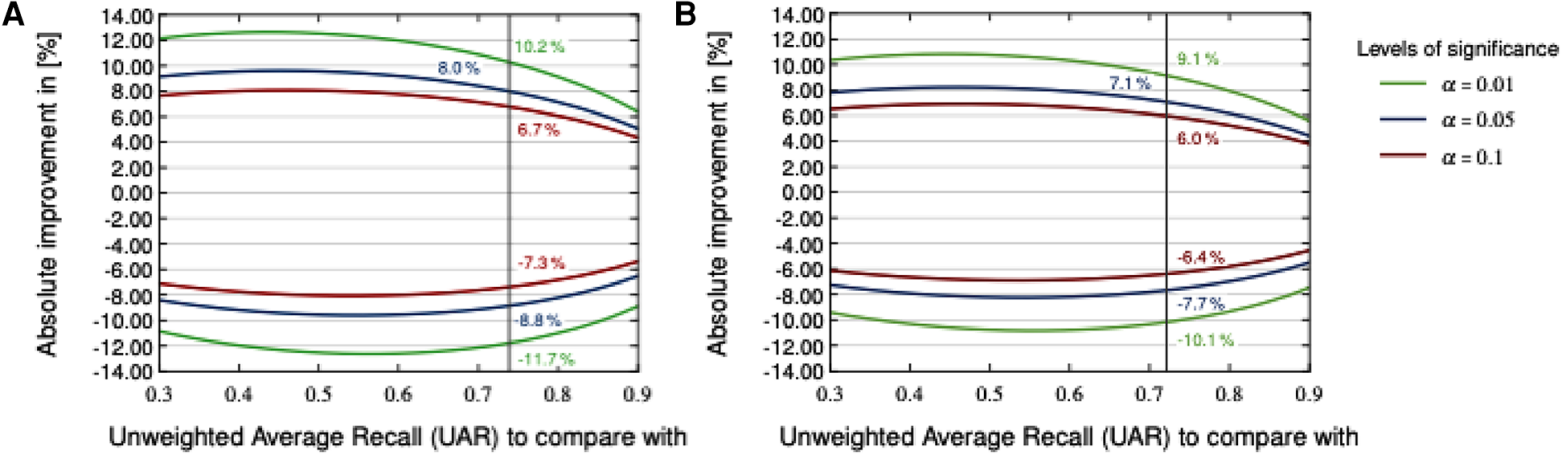
Two-sided significance test on the COVID-19 Cough (**A**) and Speech (**B**) test sets with various levels of significance according to a two-sided Z-test.

Figures 2 and 3 detail the rankings for the 19 teams which submitted predictions for the test set. We congratulate (23) for winning the COVID-19 Cough Sub-Challenge with an UAR of 75.9% on the held out test set.^2^ We note that for the COVID-19 Speech Sub-Challenge, no team exceeded the performance of the baseline which scored 72.1% UAR on the held out test set. To significantly outperform the baseline system for the cough modality, with a significance level of α=0.1, as detailed in Figure 4, would require an improvement of 6.7%, an improvement which the winning submission fell short of by 4.7%.

For both Sub-Challenges, teams struggled to outperform the baseline. Postulating why this could be the case one could suggest one, or a combination, of the following: COVID-19 detection from audio is a particularly hard task, the baseline score—being already a fusion of several state-of-the-art systems for CCS—represents a performance ceiling and that higher classification scores are not possible for this dataset, or, as a result of the limited size of the dataset, the task lends itself to less data hungry algorithms, such as the openSMILE-SVM baseline models for CSS.

**Figure 5:**
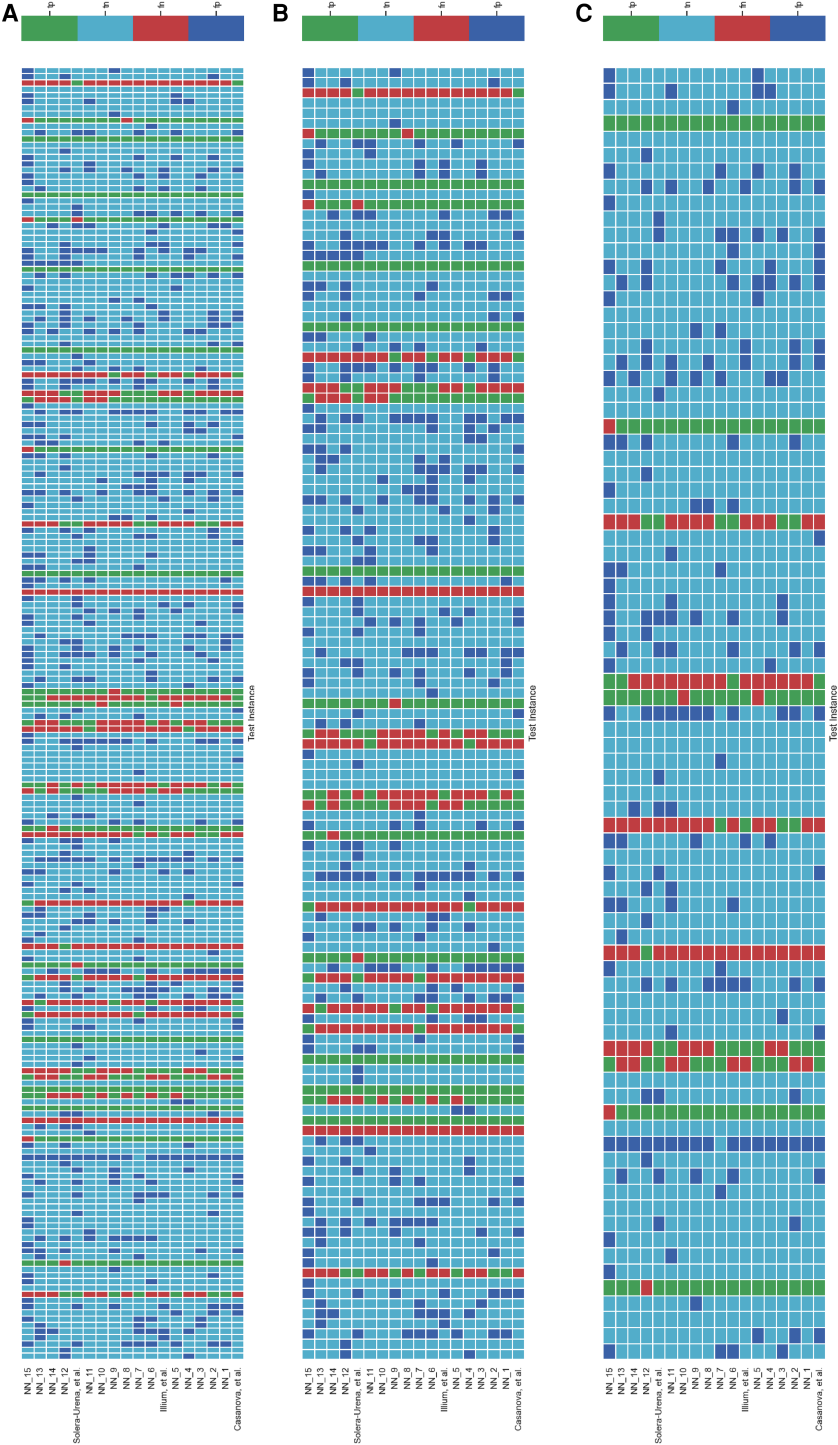
Schematic detailing the level of agreement between teams for each test instance for the **COVID-19 Cough Sub-Challenge**. Each row represents a team’s submission results. The teams have been ordered by Unweighted Average Recall, from the bottom up (team Casanova et al.’s predictions represent the highest scoring submission). Each column represents all teams predictions, across the competition, for one test instance. The test instances appear in the order in which they are in the test set. (**A**) Details all the test instances, (**B**) details only the test instances which were experiencing symptoms at the time of recording, and (**C**) details only the test instances which were experiencing no symptoms at the time of recording.

**Figure 6:**
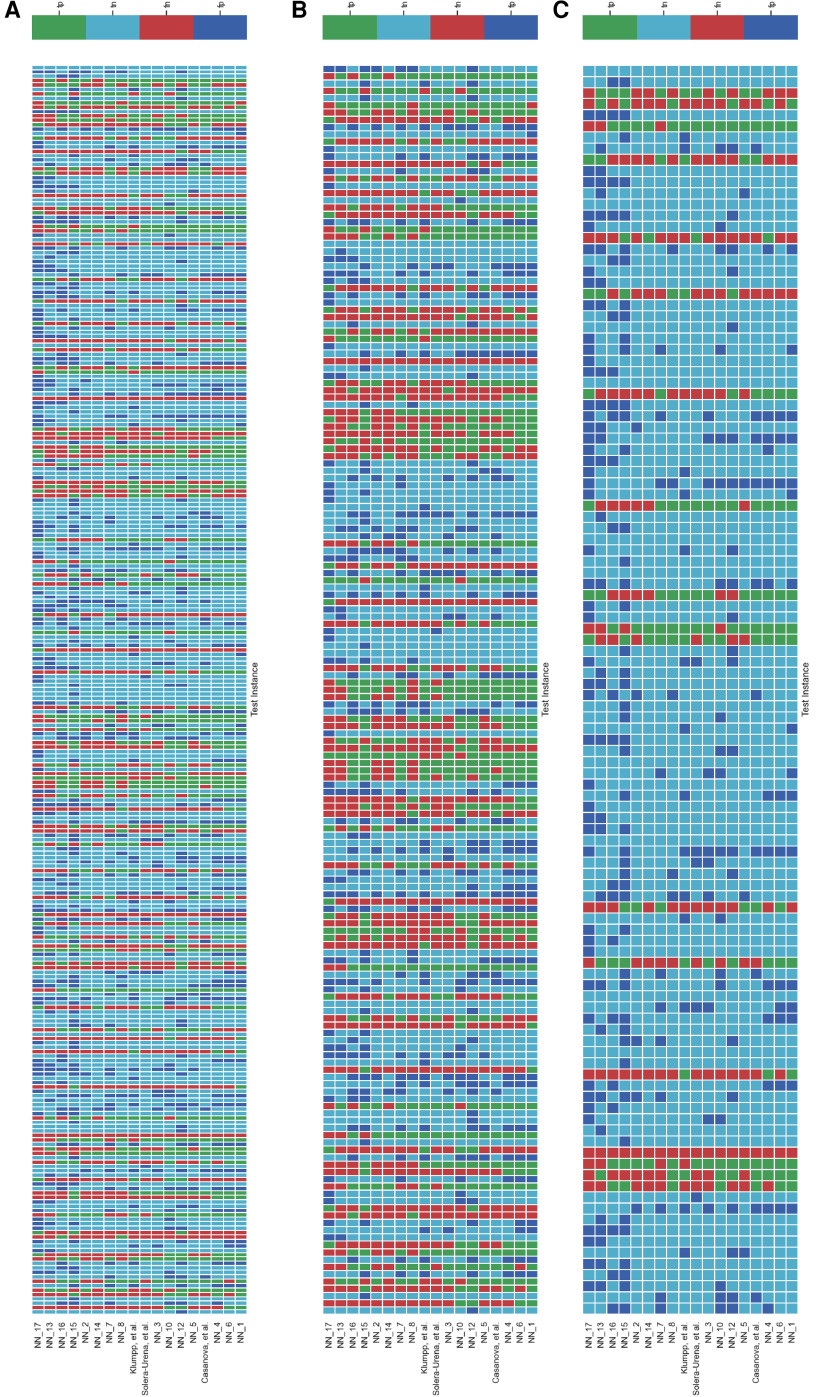
Schematic detailing the level of agreement between teams for each test instance for the **COVID-19 Speech Sub-Challenge**. Each row represents a team’s submission results. The teams have been ordered by Unweighted Average Recall (UAR), from the bottom up (team *yoshiharuyamamoto*’s predictions represent the highest scoring submission). Each column represents all teams’ predictions, across the competition, for one test instance. The test instances appear in the order which they are in the test set. note: There are more test cases in the COVID-19 Speech Sub-Challenge than in the COVID-19 Cough Sub-Challenge. (**A**) Details all the test instances, (**B**) details only the test instances which were experiencing symptoms at the time of recording, and (**C**) details only the test instances which were experiencing no symptoms at the time of recording.

It is important to analyse the level of agreement of COVID-19 detection between participant submissions. This is shown schematically in Figures 5 and 6. From these figures, we can see that there are clearly COVID-19 positive cases which teams across the board are able to correctly predict, but there are also positive COVID-19 cases which all teams have missed. These findings are reflected in the minimal performance increase of 0.3% and 0.8% for cough and speech tasks, respectively, obtained when fusing n best submission predictions through majority voting schemes. The results from fusing n best models using majority voting are detailed in Figures A2 and A3 . This suggests that models from all teams are depending on similar audio features when predicting COVID-19 positive cases.

**Figure A1:**
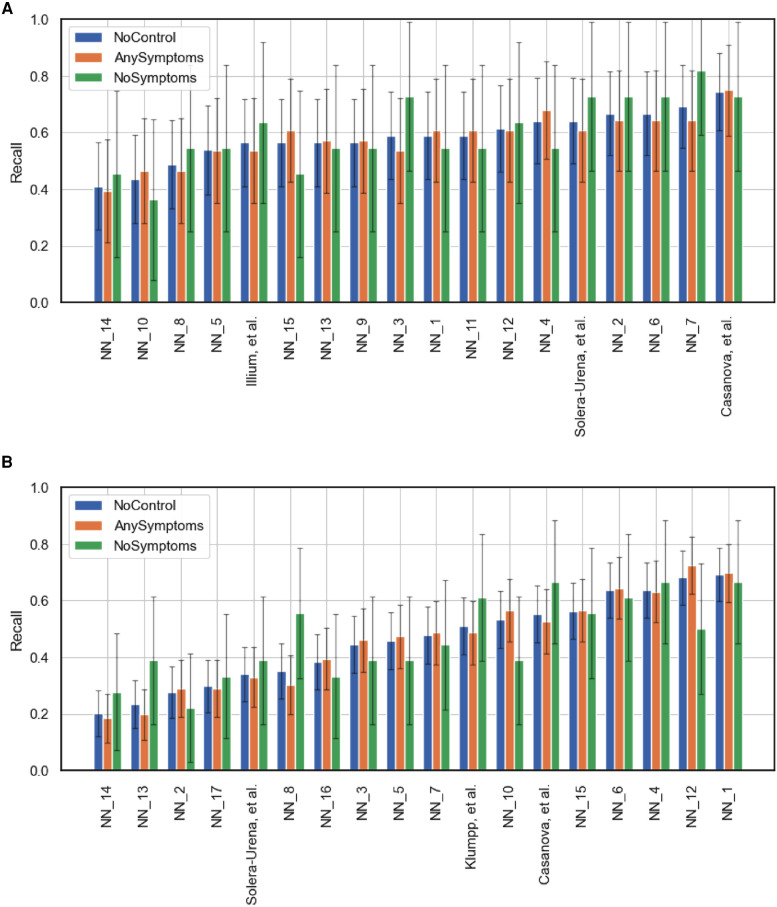
Team performance on the full test set (NoControl) and two curated test sets featuring only test instances where the participants either had at least one symptom (AnySymptoms) or were displaying no symptoms at all (NoSymptoms). The metric reported is recall for positive cases. 95% confidence intervals are shown, calculated via the normal approximation method. (**A**) Corresponds to the COVID-19 Cough Sub-Challenge, CCS, and (**B**) the COVID-19 Speech Sub-Challenge, CSS.

**Figure A2:**
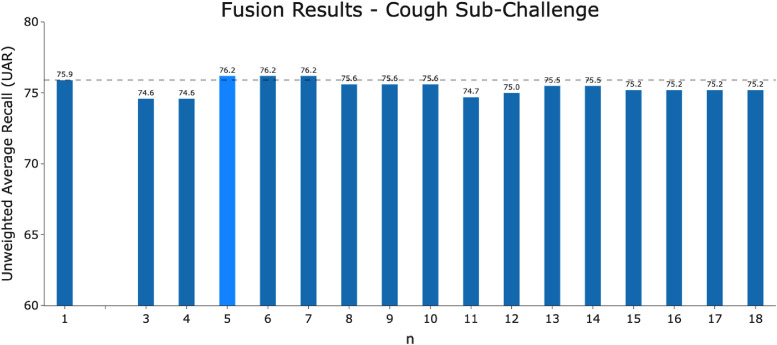
The performance of the fusion model of n-best models for the COVID-19 Cough Sub-Challenge using majority voting.

**Figure A3:**
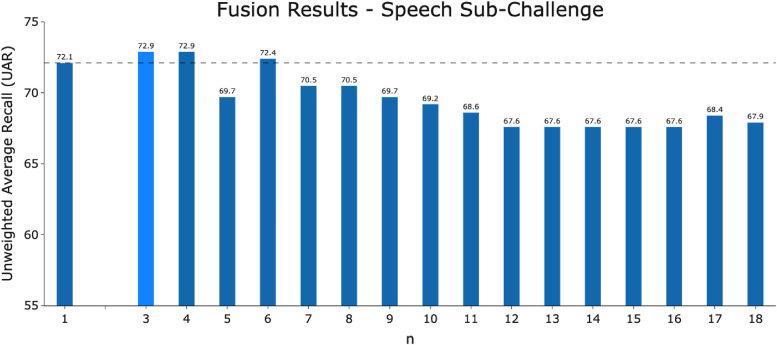
The performance of the fusion model of n-best models for the COVID-19 Speech Sub-Challenge using majority voting.

Figures 5 and 6B,C detail the level of agreement across submissions for curated subset of the test set, where participants were selected if they were displaying at least one symptom (b) and when they were displaying no symptoms (c). These figures can be paired with Figure A1 which details the recall scores for positive cases across these same curated test sets. From this analysis, it does not appear that there was a trend across teams to perform favourably on cases where symptoms were being displayed or vice versa. While this does not disprove worries that these algorithms are simply cough or symptom identifiers, it does not add evidence in support of this claim.

### Limitations

While this challenge was an important step in exploring the possibilities of a digital mass test for COVID-19, it has a number of limitations. A clear limiting factor of the challenge was the small size of the dataset. While many participants addressed this through data augmentation and regularisation techniques, it restricted the extent to which conclusions could be taken from the results, particularly investigating teams’ performance on carefully controlled subsets of the data. We look forward to the newly released COVID-19 sounds dataset (21) which represents a vastly greater source of COVID-19 samples.

**Figure A4:**
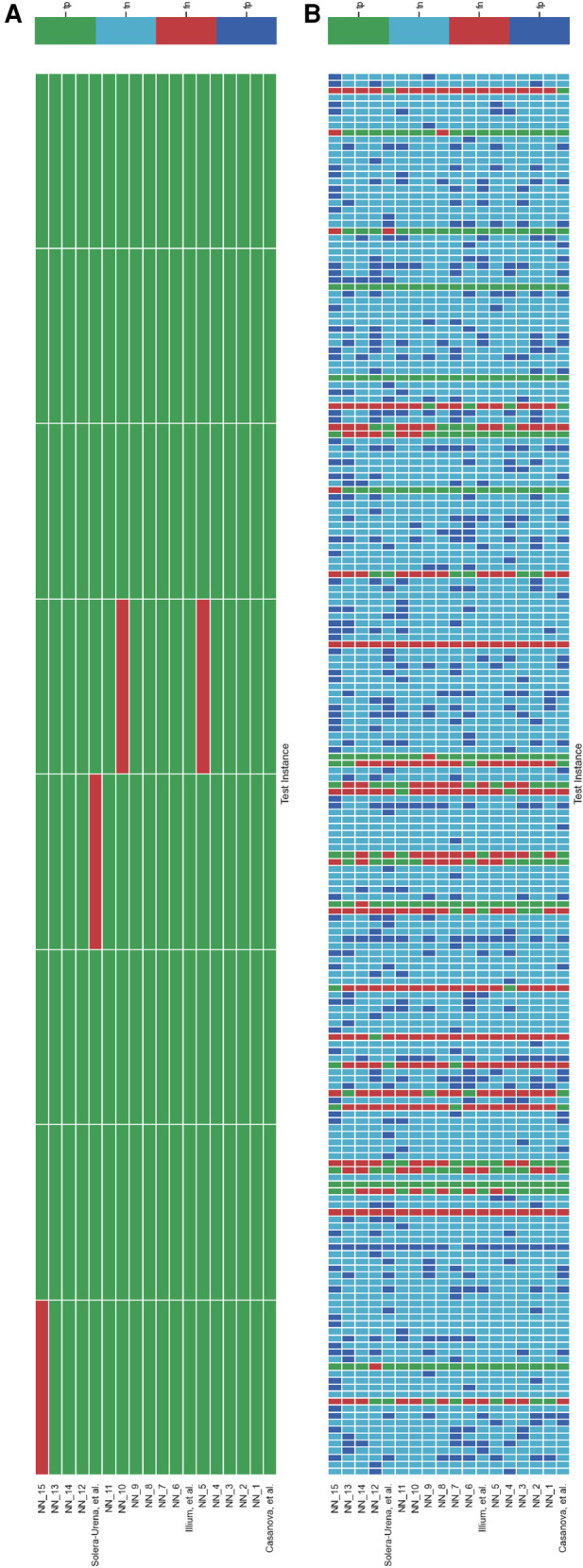
Schematic detailing the level of agreement as in Figure 5 with test instances with either a low sample rate (below 12 kHz) (**A**) or high sample rate (above 12 kHz) (**B**).

**Figure A5:**
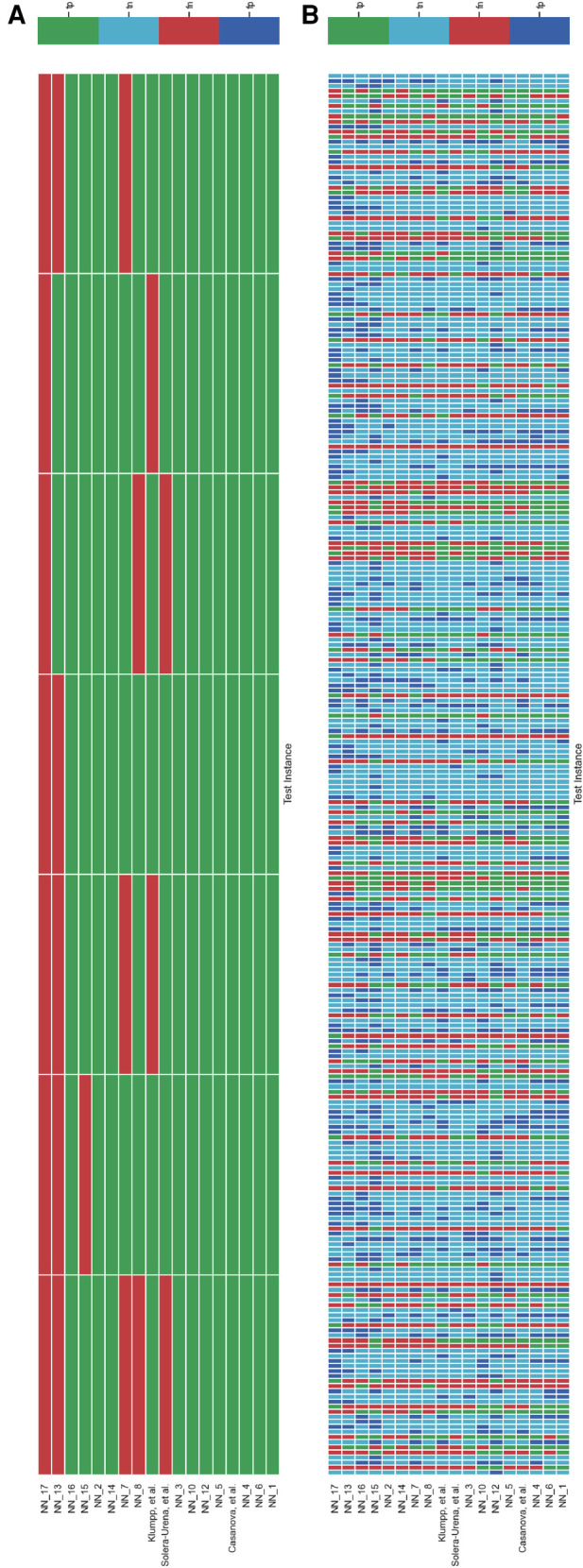
Schematic detailing the level of agreement as in Figure 6 with test instances with either a low sample rate (below 12 kHz) (**A**) or high sample rate (above 12 kHz) (**B**).

**Figure A6:**
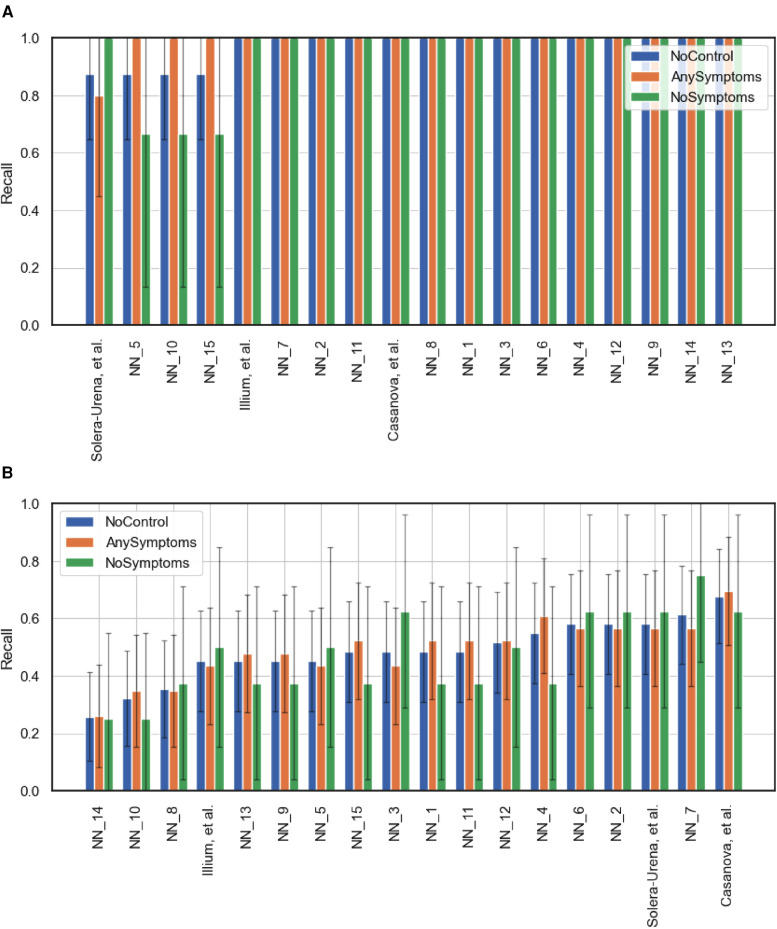
Team performance on two curated test sets from the COVID-19 Cough Sub-Challenge. (**A**) Controls for test samples with a sample rate of greater than 12 kHz and (**B**) controls for test samples with a sample rate of 12 kHz and below. The metric reported is recall for positive cases. 95% confidence intervals are shown, calculated via the normal approximation method.

**Figure A7:**
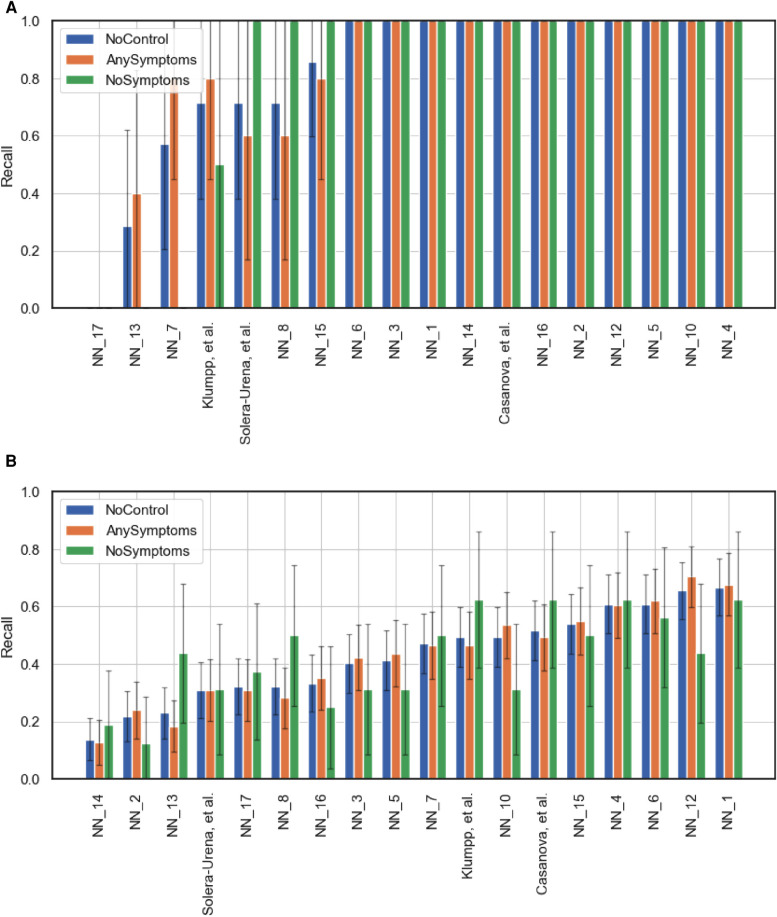
Team performance on two curated test sets from the COVID-19 Speech Sub-Challenge. (**A**) Controls for test samples with a sample rate of greater than 12 kHz and (**B**) controls for test samples with a sample rate of 12 kHz and below. The metric reported is recall for positive cases. 95% confidence intervals are shown, calculated via the normal approximation method.

A further limitation of this challenge is the unforeseen correlation between low sample rate recordings, below 12 kHz, and COVID-19 status. In fact all low sample rate recordings in the challenge for both CCS and CSS were COVID-19 positive. For CCS and CSS there were 30 and 37 low sample rate cases, respectively. The reason for this is that at the start of the study the label in the survey for COVID-19 negative was unclear, and could have been interpreted as either “not tested” or “tested negative.” For this reason no negative samples from the time period were used. This can be seen in Figure 1A. This early version of data collection also correlated with the study allowing for lower sample rate recordings, a feature which later was changed to restrict submissions to higher sample rates. This resulted in all the low sample rate recordings being COVID-19 positive. As can be seen in Figures A4, A5, A6 and A7, teams’ trained models were able to pick up on the sample rate bias, with most teams correctly predicting all the low sample rate cases as COVID-19 positive. When this is controlled for and low sample rate recordings are removed from the test set, as shown in Figures A6 and A7, teams’ performances drop significantly. For the challenge baselines this too was the case, with the fusion of baseline models for CCS falling from 73.8% to 68.6% UAR and the opensmile-SVM baseline for CSS dropping from 72.1% to 70.9% UAR. This is a great example of the effect of overlooked bias which expresses itself as an identifiable audio feature, leading to inflated classification scores. We regret that this was not found earlier. Inspecting Figure 1A) further, one will also realise that all the COVID-19 negative individuals were collected in the summer of 2020, one could argue that this ascertainment bias injected further imbalance between COVID-19 negative and positive individuals. An example of this is that individuals are much less likely to have the flu in summer (43), resulting in respiratory symptoms having an inflated correlation with COVID-19 status in the collected dataset compared to the general population. This has been shown to artificially boost model performance at COVID-19 detection (44–46). In future more factors, which can be a source of bias, should be controlled for, namely in this case, age of participant, gender, symptoms, location of recording and date of recording. Matching on these attributes would yield more realistic performance metrics.

As with most machine learning methods, it still remains unclear how to interpret the decision making process at inference time. This results in it being tricky to determine which acoustic features the model is correlating with COVID-19. Whether that be true, acoustic features caused by the COVID-19 infection or other acoustic bias (15, 44). We also note that this is a binary classification task, in that models only had to decide between COVID-19 positive or negative. This “closed word fallacy” (42) leads to inflated performance as models are not tasked with discerning between confounding symptoms such as heavy cold or asthma. Tasking models to predict COVID-19 out of a wide range of possible conditions/symptoms would be a harder task. The test set provided saw a complete temporal overlap with the training set, in future it would be nice to experiment with time disjoint test sets, as in (44) to investigate whether the signal for COVID-19 changes over time. Collecting a dataset which yields a test set with a higher proportion of COVID positive individuals is also desirable.

In this challenge, participants were provided with the test set recordings (without the corresponding labels). In future challenges, test set instances should be kept private, requiring participants to submit trained models along with pipeline scripts for inference. Teams’ test set predictions can then be run automatically by the challenge organisers. This will help in reducing the possibility of overfitting and foul play. We note that there was no evidence of foul play, e.g., training in an unsupervised manner on the test set, in this challenge.

Another limitation of this challenge was the lack of meta data that organisers could provide to participants. This tied teams’ hands to some extent in evaluating for themselves the level of bias in the dataset and so their opportunity to implement methods to combat it. This was not a desired feature. However, we now point teams towards the newly open sourced COVID-19 Sounds database (21) which also provides collected meta data. It is this dataset from which a subset of samples was taken for this challenge.

## Conclusion

This challenge demonstrated that there is a signal in crowdsourced COVID-19 respiratory sounds that allows for machine learning algorithms to fit a classifier which achieves moderate detection rates of COVID-19 in infected individuals’ respiratory sounds. Exactly what this signal is, however, still remains unclear. Whether these signals are truly audio biomarkers in respiratory sounds of infected individuals uniquely caused by COVID-19 or rather identifiable bias in the datasets, such as confounding flu like symptoms, is still an open question to be answered next.

## Data Availability

The original contributions presented in the study are included in the article/supplementary material, further inquiries can be directed to the corresponding author/s.

## Footnotes

^1^ https://dicova2021.github.io

^2^UAR is the established ComParE evaluation metric. UAR is equivalent to balanced accuracy. We note that if F1 had been the evaluation metric, Illium, et al. (25) would have infact won the cough sub challenge. This is thanks to their model’s superior precision performance, i.e., what proportion of the model’s positive predictions are correct.

## Appendix

Here we present some results from ablation studies of teams’ performances through evaluating performance on curated subsets of the test set. Figure A1 details the effect of controlling for symptom cofounders on teams’ performance. Figures A6 and A7 repeats this analysis however controlling for sample rate. Figures A4 and A5 details the level of agreement between teams for the low Figures A4A, A5A and high A4B, A5B sample rate test cases. Figures A2 and A3 detail the classification performance of a fusion of teams’ predictions on the test set.

## References

[1] Brown C Chauhan J Grammenos A Han J Hasthanasombat A Spathis D, et al. Exploring automatic diagnosis of COVID-19 from crowdsourced respiratory sound data. In *ACM SIGKDD International Conference on Knowledge Discovery & Data Mining*; New York, NY, USA. Association for Computing Machinery (2020). p. 3474–84. Available from: 10.1145/3394486.3412865.

[2] Xia T Han J Qendro L Dang T Mascolo C. Uncertainty-aware COVID-19 detection from imbalanced sound data. *arXiv*. (2021) [preprint]. 10.48550/ARXIV.2104.02005

[3] Imran A Posokhova I Qureshi HN Masood U Riaz MS Ali K, et al. AI4COVID-19: AI enabled preliminary diagnosis for COVID-19 from cough samples via an app. Inform Med Unlocked (2020) 20:100378. 10.1016/j.imu.2020.100378 32839734 PMC7318970

[4] Sharma N Krishnan P Kumar R Ramoji S Chetupalli SR Ghosh NRPK, et al. Coswara—a database of breathing, cough,, voice sounds for COVID-19 diagnosis. In *Proceedings INTERSPEECH 2020*; Shanghai, China. ISCA (2020). p. 4811–15. Available from: 10.21437/Interspeech.2020-2768

[5] Bagad P Dalmia A Doshi J Nagrani A Bhamare P Mahale A, et al. Cough against COVID: evidence of COVID-19 signature in cough sounds. *arXiv* (2020) [preprint]. 10.48550/arXiv.2009.08790

[6] Pinkas G Karny Y Malachi A Barkai G Bachar G Aharonson V. SARS-CoV-2 detection from voice. Open J Eng Med Biol (2020) 1:268–74. 10.1109/OJEMB.2020.3026468 PMC8769003 35402954

[7] Orlandic L Teijeiro T Atienza D. The COUGHVID crowdsourcing dataset, a corpus for the study of large-scale cough analysis algorithms. Sci Data (2021) 8:156. 10.1038/s41597-021-00937-4 34162883 PMC8222356

[8] Andreu-Perez J Perez-Espinosa H Timonet E Kiani M Giron-Perez MI Benitez-Trinidad AB, et al. A generic deep learning based cough analysis system from clinically validated samples for point-of-need COVID-19 test, severity levels. IEEE Trans Serv Comput (2021). 10.1109/TSC.2021.3061402 PMC9328729 35936760

[9] Pizzo DT Santiago Esteban S de los Ángeles Scetta M. IATos: AI-powered pre-screening tool for COVID-19 from cough audio samples. *arXiv*. (2021) [preprint]. 10.48550/arXiv.2104.13247

[10] Qian K Schmitt M Zheng H Koike T Han J Liu J, et al. Computer audition for fighting the SARS-CoV-2 corona crisis – introducing the multi-task speech corpus for COVID-19. Internet Things J (2021) 8(21):16035–46. 10.1109/JIOT.2021.3067605 PMC8768988 35782182

[11] Bartl-Pokorny KD Pokorny FB Batliner A Amiriparian S Semertzidou A Eyben F, et al. The voice of COVID-19: acoustic correlates of infection in sustained vowels. J Acoust Soc Am (2021) 149:4377–83. 10.1121/10.0005194 34241490 PMC8269757

[12] Coppock H Gaskell A Tzirakis P Baird A Jones L Schuller BW. End-to-end convolutional neural network enables COVID-19 detection from breath, cough audio: a pilot study. BMJ Innov (2021) 7:356–62. 10.1136/bmjinnov-2021-000668 34192022

[13] Nessiem MA Mohamed MM Coppock H Gaskell A Schuller BW. Detecting COVID-19 from breathing, coughing sounds using deep neural networks. In *International Symposium on Computer-Based Medical Systems (CBMS)*; Aveiro, Portugal. IEEE (2021). p. 183–8. Available from: 10.1109/CBMS52027.2021.00069.

[14] Ponomarchuk A Burenko I Malkin E Nazarov I Kokh V Avetisian M, et al. Project achoo: a practical model and application for COVID-19 detection from recordings of breath, voice, and cough. IEEE J Sel Top Signal Process (2022) 16(2):175–87. 10.1109/JSTSP.2022.3142514 35582703 PMC9088778

[15] Coppock H Jones L Kiskin I Schuller BW. COVID-19 detection from audio: seven grains of salt. Lancet Digit Health (2021) 3(9):e537–8. 10.1016/S2589-7500(21)00141-2 34303644 PMC8294804

[16] Han J Xia T Spathis D Bondareva E Brown C Chauhan J, et al. Sounds of COVID-19: exploring realistic performance of audio-based digital testing. *NPJ Digit Med*. (2022) 28:5(1). 10.1038/s41746-021-00553-x PMC8799654 35091662

[17] Coppock H Jones L Kiskin I Schuller BW. Bias and privacy in AI’s cough-based COVID-19 recognition – Authors’ reply. Lancet Digit Health (2021) 3:e761. 10.1016/S2589-7500(21)00233-8 34823704 PMC8608389

[18] Akman A Coppock H Gaskell A Tzirakis P Jones L Schuller BW. Evaluating the COVID-19 identification ResNet (CideR) on the INTERSPEECH COVID-19 from audio challenges. *Front Digit Health*. (2022) 4:789980. 10.3389/fdgth.2022.789980 PMC9302571 35873349

[19] Schuller BW Batliner A Bergler C Mascolo C Han J Lefter I, et al. The interspeech 2021 computational paralinguistics challenge: COVID-19 cough, COVID-19 speech, escalation & primates. In *INTERSPEECH 2021, 22nd Annual Conference of the International Speech Communication Association*; Brno, Czechia (2021).

[20] Muguli A Pinto L Sharma NRN Krishnan P Ghosh PK Kumar R, et al. DiCOVA challenge: dataset, task, and baseline system for COVID-19 diagnosis using acoustics. *arXiv*. (2021) 10.48550/arXiv.2103.09148

[21] Xia T Spathis D Brown C Ch AGJ Han J Hasthanasombat A, et al. COVID-19 sounds: a large-scale audio dataset for digital COVID-19 detection. *NeurIPS 2021 Track Datasets and Benchmarks Round2 Submission*. OpenReview (2021). Available from: https://openreview.net/forum?id=9KArJb4r5ZQ.

[22] Solera-Ureña R Botelho C Teixeira F Rolland T Abad A Trancoso I. Transfer learning-based cough representations for automatic detection of COVID-19. In *Proceedings of Interspeech 2021* (2021). p. 436–40. Available from: 10.21437/Interspeech.2021-1702.

[23] Casanova E Candido Jr A Fernandes Jr RC Finger M Gris LRS Ponti MA, et al. Transfer learning and data augmentation techniques to the COVID-19 identification tasks in ComParE 2021. In *Proceedings of Interspeech 2021* (2021). p. 446–50. Available from: 10.21437/Interspeech.2021-1798.

[24] Klumpp P Bocklet T Arias-Vergara T Vásquez-Correa J Pérez-Toro P Bayerl S, et al. The phonetic footprint of COVID-19? In *Proceedings of Interspeech 2021* (2021). p. 441–5. Available from: 10.21437/Interspeech.2021-1488.

[25] Illium S Müller R Sedlmeier A Popien C-L. Visual transformers for primates classification and covid detection. In *Proceedings of Interspeech 2021* (2021). p. 451–455. Available from: https://doi.ord/10.21437/Interspeech.2021-273.

[26] Schuller B Steidl S Batliner A. The INTERSPEECH 2009 emotion challenge. In *Proceedings of INTERSPEECH*; Brighton. (2009). p. 312–5.

[27] Manning CD Raghavan P Schútze H, An introduction to information retrieval. Cambridge: Cambridge University Press (2009).

[28] Eyben F Weninger F Gross F Schuller B. Recent developments in opensmile, the munich open-source multimedia feature extractor. In *Proceedings of the 21st ACM International Conference on Multimedia*, MM ’13; New York, NY, USA. Association for Computing Machinery (2013). p. 835–8. Available from: 10.1145/2502081.2502224

[29] Schmitt M Schuller B. openXBOW – introducing the passau open-source crossmodal bag-of-words toolkit. J Mach Learn Res. (2017) 18:3370–4.

[30] Amiriparian S Gerczuk M Ottl S Cummins N Pugachevskiy S Schuller B. Bag-of-deep-features: noise-robust deep feature representations for audio analysis. In *2018 International Joint Conference on Neural Networks (IJCNN)* (2018). p. 1–7. Available from: 10.1109/IJCNN.2018.8489416.

[31] Freitag M Amiriparian S Pugachevskiy S Cummins N Schuller B. auDeep: unsupervised learning of representations from audio with deep recurrent neural networks. J Mach Learn Res. (2018) 18:6340–44.

[32] Villalba J Chen N Snyder D Garcia-Romero D McCree A Sell G, et al. State-of-the-art speaker recognition with neural network embeddings in NIST SRE18 and speakers in the wild evaluations. Comput Speech Lang (2020) 60:101026. 10.1016/j.csl.2019.101026

[33] Hershey S Chaudhuri S Ellis DPW Gemmeke JF Jansen A Moore RC, et al. Cnn architectures for large-scale audio classification. New Orleans, LA, United States: 017 IEEE International Conference on Acoustics, Speech and Signal Processing (ICASSP) (2017). p. 131-5. 10.1109/ICASSP.2017.7952132

[34] Ravanelli M Zhong J Pascual S Swietojanski P Monteiro J Trmal J, et al. Multi-task self-supervised learning for robust speech recognition. Barcelona, Spain: ICASSP 2020 - 2020 IEEE International Conference on Acoustics, Speech and Signal Processing (ICASSP) (2020). p. 6989–93. 10.1109/ICASSP40776.2020.9053569

[35] Tzirakis P Zafeiriou S Schuller BW. End2you – the imperial toolkit for multimodal profiling by end-to-end learning. *arXiv* (2018) [preprint]. 10.48550/arXiv.1802.01115

[36] Casanova E Gris L Camargo A da Silva D Gazzola M Sabino E, et al. Deep learning against COVID-19: respiratory insufficiency detection in Brazilian Portuguese speech. In *Findings of the Association for Computational Linguistics: ACL-IJCNLP 2021*. Association for Computational Linguistics (2021). p. 625–33. Available from: 10.18653/v1/2021.findings-acl.55.

[37] Kong Q Cao Y Iqbal T Wang Y Wang W Plumbley MD. PANNs: large-scale pretrained audio neural networks for audio pattern recognition. IEEE/ACM Transactions on Audio, Speech, and Language Processing, vol. 28 (2020). p. 2880–94. 10.1109/TASLP.2020.3030497

[38] Dosovitskiy A Beyer L Kolesnikov A Weissenborn D Zhai X Unterthiner T, et al. An image is worth 16×16 words: transformers for image recognition at scale. *arXiv*. (2021) [preprint]. 10.48550/arXiv.2010.11929

[39] Akiba T Sano S Yanase T Ohta T Koyama M. Optuna: a next-generation hyperparameter optimization framework. CoRR. (2019) [preprint]. 10.48550/arXiv.1907.10902

[40] Isaac E. Test of hypothesis - concise formula summary (2015).

[41] Wasserstein RL Lazar NA. The ASA’s statement on p-values: context, process, and purpose. Am Stat (2016) 70:129–33. 10.1080/00031305.2016.1154108

[42] Batliner A Hantke S Schuller B. Ethics and good practice in computational paralinguistics. IEEE Trans Affect Comput. (2020). 13(3):1236–53. 10.1109/TAFFC.2020.3021015

[43] Lowen AC Steel J. Roles of humidity and temperature in shaping influenza seasonality. J Virol (2014) 88(14):7692–5. 10.1128/JVI.03544-13 24789791 PMC4097773

[44] Coppock H Nicholson G Kiskin I Koutra V Baker K Budd J, et al. Audio-based ai classifiers show no evidence of improved COVID-19 screening over simple symptoms checkers. *arXiv*. (2022) [preprint]. 10.48550/arXiv.2212.08570

[45] Pigoli D Baker K Budd J Butler L Coppock H Egglestone S, et al. Statistical design and analysis for robust machine learning: a case study from COVID-19. *arXiv*. (2022) [preprint]. 10.48550/arXiv.2212.08571

[46] Budd J Baker K Karoune E Coppock H Patel S Cañadas AT, et al. A large-scale and PCR-referenced vocal audio dataset for COVID-19. *arXiv*. (2022) [preprint]. 10.48550/arXiv.2212.07738 PMC11211414 38937483

